# Customizable orthopaedic oncology implants: one institution’s experience with meeting current IRB and FDA requirements

**DOI:** 10.1186/s40064-016-2696-1

**Published:** 2016-07-04

**Authors:** Alexander R. Willis, Joseph A. Ippolito, Francis R. Patterson, Joseph Benevenia, Kathleen S. Beebe

**Affiliations:** Monmouth Medical Center, Long Branch, NJ 07740 USA; Department of Orthopaedic Surgery, Rutgers New Jersey Medical School, 140 Bergen Street, ACC Building, Suite D-1610, Newark, NJ 07103 USA

## Abstract

**Background:**

Customizable orthopaedic implants are often needed for patients with primary malignant bone tumors due to unique anatomy or complex mechanical problems. Currently, obtaining customizable orthopaedic implants for orthopaedic oncology patients can be an arduous task involving submitting approval requests to the Institutional Review Board (IRB) and the Food and Drug Administration (FDA). There is great potential for the delay of a patient’s surgery and unnecessary paperwork if the submission pathways are misunderstood or a streamlined protocol is not in place.

**Purpose:**

The objective of this study was to review the existing FDA custom implant approval pathways and to determine whether this process was improved with an institutional protocol.

**Methods:**

An institutional protocol for obtaining IRB and FDA approval for customizable orthopaedic implants was established with the IRB at our institution in 2013. This protocol was approved by the IRB, such that new patients only require submission of a modification to the existing protocol with individualized patient information. During the two-year period of 2013–2014, eight patients were retrospectively identified as having required customizable implants for various orthopaedic oncology surgeries. The dates of request for IRB approval, request for FDA approval, and total time to surgery were recorded, along with the specific pathway utilized for FDA approval.

**Results:**

The average patient age was 12 years old (7–21 years old). The average time to IRB approval of a modification to the pre-approved protocol was 14 days (7–21 days). Average time to FDA approval after submission of the IRB approval to the manufacturer was 12.5 days (7–19 days). FDA approval was obtained for all implants as compassionate use requests in accordance with Section 561 of the Federal Food Drug and Cosmetic Act’s expanded access provisions.

**Conclusions:**

Establishment of an institutional protocol with pre-approval by the IRB can expedite the otherwise time-consuming and complicated process of obtaining customizable orthopaedic implants for orthopaedic oncology patients.

**Level of evidence:**

Retrospective case series, Level IV. See the Guidelines for authors for a complete description of levels of evidence.

## Background

Malignant primary bone tumors are rare entities with an incidence of approximately 0.8/100,000 people per year. In the adult population, primary malignant bone tumors represent 0.2 % of all tumors, whereas in the pediatric population, they account for approximately 5 % of all malignancies (Kindblom 2009; Dorfman and Czerniak 1995). The three most common malignant primary bone tumors are osteosarcoma, chondrosarcoma, and Ewing’s sarcoma, which combined, represent 75 % of all malignant primary bone tumors (Dorfman and Czerniak 1995). In the US, osteosarcoma is most common with an incidence of approximately 400 cases per year, holding the sixth highest prevalence of all cancers in children less than fifteen years of age (Mirabello et al. 2009).

When a malignant primary bone tumor is diagnosed, patients often require extensive surgery and removal of large portions of their skeletal structure along with the tumor. Historically, amputation was once the mainstay of treatment of these tumors. However, more recent advances have made limb salvage surgery feasible (Lewis 1985; Link et al. 1986). These procedures require significant pre-operative planning, part of which includes obtaining an implant appropriate for the patient’s size, anatomy, and defect created by the surgery.

Due to the predilection of osteosarcoma for the distal femur, proximal tibia, and proximal humerus, limb length discrepancy can be severe following tumor and concomitant physeal resection (Ottaviani and Jaffe 2009). The proximal humerus contributes approximately 80 % to the final humeral length, the distal femur contributes approximately 70 % to the final femoral length, and the proximal tibia contributes approximately 57 % to the final tibial length (Tsuchihara et al. 2008; Pritchett 1992).

Over the last 2–3 decades, expandable prostheses have supplanted older devices due to their ability to simultaneously reconstruct the limb and address potential limb length discrepancies that may occur in skeletally immature patients following physeal resection (Eckardt et al. 2000; Finn and Simon 1991). Implantation of an expandable prosthesis is indicated for limb-salvage in skeletally immature patients in which wide resection includes removal of an active physis and the patient is left with a projected limb-length discrepancy of ≥6 cm (Harvey et al. 2010; Holm et al. 1994; Papaioannou et al. 1982; Song et al. 1997; Stanitski 1999). Growth remaining was determined according to the standard methods after bone age was assessed (Anderson et al. 1963; Dimeglio 2001). For use in the humerus, the Repiphysis^®^ was discussed with the patient and family as a limb-salvage option. As patient size, age, anatomy, and location of tumor vary greatly, approved off-the-shelf implants are not always available to fit the patient’s needs. Additionally, despite the progress that has been made with respect to the design of orthopaedic devices, mechanical failure is not uncommon. Failure of one or more components of an implanted system may leave the surgeon with a unique situation that demands either customization or revision components or importation of devices used in other parts of the world, which are inherently not FDA approved. All devices used in the United States require FDA approval prior to implantation, and therefore, this creates a distinct logistical challenge for the surgeon.

### Pathway overview

The FDA has multiple pathways (Table 1) in place to facilitate justified and expeditious acquisition of safe and effective implants surgeons or dentists may need. Nevertheless, without proper guidance, it has become somewhat onerous to obtain the implants required, partially because of recent increases in manufacturer scrutiny. Following a Department of Justice investigation in September 2007, four major orthopaedic companies were charged with violating anti-kickback statutes and were forced into short-term intense federal monitoring. This may have inhibited willingness of manufacturers to produce custom devices out of residual concern for FDA inquiry and assessment of corporate compliance. Additionally, some surgeons may lack understanding of the FDA protocols and when each pathway is applicable. Summarized below are the primary pathways relevant to obtaining custom orthopaedic surgical implants.

**Table 1:** Overview of FDA pathways

Pathway	FDA codes	Overview	Details
Compassionate use request	21 CFR 812.35-36 and Section 561 of the FD&C Act	For use of unapproved devices from clinical trials in patients that do not meet the study’s inclusion criteria	This pathway arose as part of the “Expanded Access” provision in the FDA Modernization Act of 1997. A compassionate use request allows for investigational devices to be used in emergency situations or for patients that do not meet a clinical trial’s inclusion criteria. FDA approval is required prior to implementation and can be obtained by having the sponsor submit an Investigational Device Exemption (IDE) supplement, including a description of why treatment is needed, why alternatives are unsatisfactory, any deviations from clinical protocol, as well as patient protection measures such as IRB approval, institutional clearance, informed consent, authorization from the IDE sponsor, and independent assessment by an uninvolved physician
Custom device exemption (CDE)	21 CFR 812.3(b) and Section 520(b) of the FD&C Act	For use of a customizable device due to the rarity of a patient’s medical condition or special need	This pathway arose in 1976 as an amendment to the Federal Food, Drug, and Cosmetic Act, and was expanded in 2012 under the FDA’s Safety and Innovation Act. Use of customizable devices “should represent a narrow category for which, due to the rarity of a patient’s medical condition or physician’s special need, compliance with premarket review requirements and performance standards under Sections 514 and 515 of the FD&C Act is impractical.” The device must be created in order to comply with an order of an individual physician, sufficiently unique that clinical investigation or performance standards would not apply. The customizable implant must be produced in quantities of less than 5 per year per device per patient
Humanitarian use device (HUD)	FD&C Section 520 (m)	For use of a device in treatment of a diagnosis which manifests in less than 4000 individuals per year in the United States	This pathway allows for exemption of scientifically based validation studies of efficacy mandated for standard premarket approval as long as there is enough information to suggest that the benefit is probably greater than the risk and no better alternative exists. Once a manufacturer has obtained an HDE approval for a specific device, IRB approval must be obtained from individual institutions, which may result in institutional device approval or necessitate IRB approval on a case-by-case basis
Premarket notification and premarket approval application	21 CFR 807.92(a)(3),862.9, 21 CFR 864.9	For devices to be used prior to marketing and sale	To obtain premarket approval for marketing and sale, there must be valid scientific evidence demonstrating the product is safe and effective. For premarket notification [510(k)], there must be evidence demonstrating a device is as safe as a currently legally marketed device. Many orthopaedic and implantable cardiac devices are approved via supplemental PMA pathways

### Compassionate use request

The FDA normally allows for unapproved, investigational devices to be used in clinical trials with specific criteria and specific protocols, under an Investigational Device Exemption (IDE). However, it is also possible to use a device currently under investigation, but outside of the clinical trial, in order to help a patient with a serious or life-threatening condition under 21 CFR 812.35-36 and Section 561 of the FD&C Act (Investigational Device Exemption). Emergency use of unapproved devices is allowed, but the sponsor (responsible party) must notify the FDA within 5 days following the procedure. The “compassionate use” request is a helpful pathway that allows physicians to use unapproved devices from clinical trials on patients that do not meet the study’s inclusion criteria, but will benefit from the device. This is also known as the “Expanded Access” provision, which was included in the FDA Modernization Act of 1997. FDA approval is required prior to implementation of the device and can be obtained by having the sponsor submit an IDE supplement including a description of why treatment is needed, why alternatives are unsatisfactory, any deviations from the clinical protocol, and patient protection measures such as IRB approval, institutional clearance, informed consent, authorization from the IDE sponsor, and independent assessment by an uninvolved physician (Investigational Device Exemption 2014).

### Custom device exemption

The Custom Device Exemption (CDE) pathway has been around since the 1976 amendments to the Federal Food, Drug, and Cosmetic Act (FD&C) (Mihalko 2015). The pathway was expanded in 2012, under the Food and Drug Administration Safety and Innovation Act (FDASIA) to allow for more flexibility in approving devices, but also to require annual industry reporting policy (Mihalko 2015). The guidance document describes that the use of custom devices “should represent a narrow category for which, due to the rarity of a patient’s medical condition or physician’s special need, compliance with premarket review requirements and performance standards under Sections 514 and 515 of the FD&C Act is impractical.” The pathway mandates that to be considered a custom device, the device must be created in order to comply with an order of an individual physician, sufficiently unique that clinical investigation or performance standards would not apply, not generally available in the US, designed for unique pathology, manufactured on case-by-case basis or for a unique subset, and produced in quantities of less than five per year [this is specified in Section 520(b) of the FD&C Act and at 21 CFR 812.3(b)]. The five units per year specification refers to five custom units of a particular type as allowed for production by a manufacturer per year. For example, a manufacturer would be allowed to produce five patient-specific custom implants of a particular device type per year. A possible scenario provided in the guidance document for appropriate use of this pathway describes a patient with skeletal dysplasia requiring a total hip replacement for osteoarthritis. A custom implant is needed due to the patient’s “unique pathological anatomy.”

### Humanitarian use device

A humanitarian use device (HUD) is defined as “a medical device intended to benefit patients in the treatment or diagnosis of a disease or condition that affects or is manifested in fewer than 4000 individuals in the United States per year.” A manufacturer of an HUD can be exempt from scientifically based validation studies of efficacy mandated for standard premarket approval as long as there is enough information to suggest that the benefit is probably greater than the risk and no better alternative exists [FD&C Section 520 (m)]. This is known as the Humanitarian Device Exemption (HDE). Once a manufacturer has obtained an HDE approval for a specific device, IRB approval must be obtained from individual institutions, which may result in institutional device approval or necessitate IRB approval on a case-by-case basis.

### Premarket notification [510(k)] and premarket approval application

Another pathway the FDA provides for approval of medical devices is via premarket notification and the premarket approval process. To obtain premarket approval for marketing and sale, there must be valid scientific evidence demonstrating the product is safe and effective. For premarket notification [510(k)], there must be evidence demonstrating a device is as safe as a currently legally marketed device. Submitters must compare their device to one or more similar legally marketed devices and make and support their substantial equivalency claims. A device is considered substantially equivalent if it has the same intended use and technological characteristics as a marketed device, or if it has the same intended use and different technological characteristics that does not raise new questions of safety and effectiveness. Many orthopaedic and implantable cardiac devices are approved via supplemental PMA pathways (Rome et al. 2014; Sheth et al. 2009).

## Methods

Our institution is a tertiary referral center for patients with musculoskeletal tumors. There are three fellowship-trained orthopaedic oncologists on staff. An institutional protocol has been developed to organize and expedite the process of approving the customizable orthopaedic implants needed for our patient population. We have determined that a customizable implant is needed approximately 2–3 times per year in our patient population (Beebe et al. 2009, 2010). Our protocol has been designed to satisfy both institutional IRB and FDA requirements for customizable implants, and this protocol has been pre-approved by our institution’s IRB to expedite the overall process. Prior to this, we needed to submit a new IRB for each patient, which would often require a significant amount of paperwork. Under our current protocol, when a patient needs a customizable implant, a modification to the approved IRB protocol is submitted to the IRB for review, which includes information regarding the specific rationalization behind our request of a customizable implant and a modified consent for surgery and implant usage. Upon IRB approval, consent forms, patient specific information, and the notice of IRB approval are forwarded to the FDA by our staff or the sponsor. After FDA approval, the approval letter is provided to the IRB and the surgery is completed when the implant arrives from the manufacturer, consents are signed, and when the patient is ready from a medical and/or oncologic standpoint. This protocol has been used for 8 retrospectively identified patients between 2013 and 2014 (Table 2).

**Table 2:** Overview of patients

Pt	Age	Diagnosis	Location	Surgery	Days to IRB approval	Days to FDA approval	Days to Sx
1	19	Ewing Sarcoma	Distal Femur	Revision	13	19	44
2	21	Ewing Sarcoma	Pelvis	Revision	18	7	35
3	11	Osteosarcoma	Distal Femur	Revision	7	15	12
4	13	Osteosarcoma	Prox Humerus	Revision	10	19	15
5	7	Osteosarcoma	Prox Tibia	Primary	12	7	28
6	11	Osteosarcoma	Prox Humerus	Primary	21	11	8
7	8	Osteosarcoma	Distal Femur	Primary	9	7	15
8	8	Osteosarcoma	Prox Humerus	Primary	21	15	4

## Results

The average patient age was 12 (range 7–21) years old at the time of surgery. The pathologic diagnosis of the patients was either osteosarcoma or Ewing’s sarcoma. Half of the patients required implants for primary tumor resection and reconstruction surgeries, and half of the customizable implants were for revision surgeries. The most common reason a patient needed a customizable implant was because they needed smaller or modified Repiphysis (Microport Orthopaedics, Arlington, TN) implants due to their age and body size (4/8 patients). For patients who underwent a primary procedure, mean time to IRB modification approval was 15.75 days (9–21 days). For patients who underwent revision, the average time to IRB modification approval was 12 days (7–18 days). The time required to complete the paperwork for an IRB modification is considerably quicker than the usual time needed for a new submission. Mean time to FDA approval after submission of the IRB approval to the sponsor was 10 days (7–15 days) for primary patients and 15 days (7–19 days) for revision patients. Mean time to surgery after FDA approval was 13.75 days (4–28 days) for primary patients and 26.5 days (12–44) for revision patients. The longest time to surgery of 44 days required importation of an implant from a manufacturer in Germany.

Many of the patients underwent pre-operative chemotherapy during the time period in which the implant was being approved. FDA approval was obtained for all implants under the “compassionate use” pathway with citation of Section 561 of the FD&C act’s expanded access provisions. None of the implants were currently involved in ongoing clinical trials or had been reviewed by the FDA as part of an IDE application.

## Discussion

A large portion of our demand for customizable implants came from pediatric patients and there was particular need for the Repiphysis Limb Salvage System (MicroPort Orthopaedics, Arlington, TN). This system has been in use since the early 1990s in Europe and has had generally good to excellent results despite a relatively high complication rate (Gitelis et al. 2003; Saghieh et al. 2010). Despite its design to address potential limb length discrepancies in skeletally immature patients, we have encountered problems requiring requesting customized Repiphysis implants that were not previously FDA approved. Two of our younger patients were too small for their lower extremity implant sizes, one patient needed modification of the FDA approved femoral Repiphysis due to inadequate femoral bone stock, and two pediatric patients needed Repiphysis implants designed for humeral tumors, which are not FDA approved, requiring utilization of the compassionate use pathway. This is representative of a longstanding deficiency in the pediatric medical device market, especially for rare pediatric conditions. This has primarily been due to insufficient financial incentives for companies potentially interested in development. A major step forward occurred in 2007 when the Pediatric Medical Device Safety and Improvement Act was passed to improve post-market surveillance and eliminate profit restrictions on HDE approved devices, in an effort stimulate production and innovation. Despite this, many pediatric devices are approved on the basis of trials conducted in non-pediatric patients (Hwang et al. 2014) and there remains significant underdevelopment in the pediatric medical device market.

The rest of the implants used required customization or modification of existing implants due to unique anatomical issues, bone loss, or a complex mechanical problem with an existing prosthesis. Because all of these situations necessitated modification of existing implants and not creation of de novo implants for unique anatomy, the “compassionate use” pathway of the Investigational Device Exemption regulation was appropriate. A de novo implant for a unique anatomical problem would be best suited by utilizing the Custom Device Exemption pathway.

Limitations in this study include the retrospective nature of this study, as well as lack of a specific control group to compare the time to IRB approval and surgery before versus after the establishment of our institutional protocol.

Obtaining the customizable orthopaedic implants necessary to promptly and properly treat patients can be complicated and time-consuming. However, with a thorough understanding of the different pathways and their indications and a protocol in place with the institution’s IRB, the process can be accelerated and less arduous for all parties involved.

## References

[CR1] Anderson M, Green WT, Messner MB (1963). Growth and predictions of growth in the lower extremities. J Bone Joint Surg Am.

[CR2] Beebe K, Song KJ, Ross E, Tuy B, Patterson F, Benevenia J (2009). Functional outcomes after limb-salvage surgery and endoprosthetic reconstruction with an expandable prosthesis: a report of 4 cases. Arch Phys Med Rehabil.

[CR3] Beebe KS, Uglialoro AD, Patel N, Benevenia J, Patterson FR (2010). Mechanical failure of the Repiphysis expandable prosthesis: a case report. J Bone Joint Surg Am.

[CR4] Dimeglio A (2001). Growth in pediatric orthopaedics. J Pediatr Orthop.

[CR5] Dorfman HD, Czerniak B (1995). Bone cancers. Cancer.

[CR6] Eckardt JJ, Kabo JM, Kelley CM, Ward WG, Asavamongkolkul A, Wirganowicz PZ, Yang RS, Eilber FR (2000). Expandable endoprosthesis reconstruction in skeletally immature patients with tumors. Clin Orthop Relat Res.

[CR7] Finn HA, Simon MA (1991). Limb-salvage surgery in the treatment of osteosarcoma in skeletally immature individuals. Clin Orthop Relat Res.

[CR8] Gitelis S, Neel MD, Wilkins RM, Rao BN, Kelly CM, Yao TK (2003). The use of a closed expandable prosthesis for pediatric sarcomas. Chir Organi Mov.

[CR9] Harvey WF, Yang M, Cooke TD, Segal NA, Lane N, Lewis CE, Felson DT (2010). Association of leg-length inequality with knee osteoarthritis: a cohort study. Ann Intern Med.

[CR10] Holm I, Nordsletten L, Steen H, Follerås G, Bjerkreim I (1994). Muscle function after mid-shaft femoral shortening. A prospective study with a two-year follow-up. J Bone Joint Surg Br.

[CR11] Hwang TJ, Kesselheim AS, Bourgeois FT (2014). Postmarketing trials and pediatric device approvals. Pediatrics.

[CR12] Investigational Device Exemption (2014) Title 21 Code of Federal Regulations Pt. 812

[CR13] Kindblom L, Davies M, Sundaram M, James S (2009). Bone Tumors: Epidemiology, Classification, Pathology. Imaging of bone tumors and tumor-like lesions.

[CR14] Lewis MM (1985). An approach to the treatment of malignant bone tumors. Orthopedics.

[CR15] Link MP, Goorin AM, Miser AW, Green AA, Pratt CB, Belasco JB, Pritchard J, Malpas JS, Baker AR, Kirkpatrick JA (1986). The effect of adjuvant chemotherapy on relapse-free survival in patients with osteosarcoma of the extremity. N Engl J Med.

[CR16] Mihalko WM (2015). How do I get what I need? Navigating the FDA’s custom, compassionate use, and HDE pathways for medical devices and implants. J Arthroplasty.

[CR17] Mirabello L, Troisi RJ, Savage SA (2009). Osteosarcoma incidence and survival rates from 1973 to 2004: data from the surveillance, epidemiology, and end results program. Cancer.

[CR18] Ottaviani G, Jaffe N (2009). The epidemiology of osteosarcoma. Cancer Treat Res.

[CR19] Papaioannou T, Stokes I, Kenwright J (1982). Scoliosis associated with limb-length inequality. J Bone Joint Surg Am.

[CR20] Pritchett JW (1992). Longitudinal growth and growth-plate activity in the lower extremity. Clin Orthop Relat Res.

[CR21] Rome BN, Kramer DB, Kesselheim AS (2014). FDA approval of cardiac implantable electronic devices via original and supplement premarket approval pathways, 1979–2012. JAMA.

[CR22] Saghieh S, Abboud MR, Muwakkit SA, Saab R, Rao B, Haidar R (2010). Seven-year experience of using Repiphysis expandable prosthesis in children with bone tumors. Pediatr Blood Cancer.

[CR23] Sheth U, Nguyen NA, Gaines S, Bhandari M, Mehlman CT, Klein G (2009). New orthopedic devices and the FDA. J Long Term Eff Med Implants.

[CR24] Song KM, Halliday SE, Little DG (1997). The effect of limb-length discrepancy on gait. J Bone Joint Surg Am.

[CR25] Stanitski DF (1999). Limb-length inequality: assessment and treatment options. J Am Acad Orthop Surg.

[CR26] Tsuchihara T, Arino H, Nemoto K, Amako M, Isaki H, Fujikawa K (2008). The growth rate of the humerus: long-term follow-up of treatment of solitary bone cyst of the proximal humerus using cannulated screws: a case report. J Pediatr Orthop B.

